# Monitoring, documenting and reporting the quality of antibiotic use in the Netherlands: a pilot study to establish a national antimicrobial stewardship registry

**DOI:** 10.1186/s12879-017-2673-5

**Published:** 2017-08-15

**Authors:** Marvin AH Berrevoets, Jaap ten Oever, Tom Sprong, Reinier M van Hest, Ingeborg Groothuis, Inger van Heijl, Jeroen A Schouten, Marlies E Hulscher, Bart-Jan Kullberg

**Affiliations:** 1 Department of Internal Medicine, Division of Infectious Diseases, Radboud university medical center, Nijmegen, The Netherlands; 2 Department of Internal Medicine, Division of Infectious Diseases, Canisius Wilhelmina Hospital, Nijmegen, The Netherlands; 3 Department of Hospital Pharmacy & Clinical Pharmacology, Academic Medical Center, Amsterdam, The Netherlands; 4 Department of Internal Medicine, Zuiderzee Medical Center, Lelystad, The Netherlands; 5 Department of Clinical Pharmacy, Tergooi Hospital, Hilversum, The Netherlands; 6 Scientific Institute for Quality of Healthcare, Radboud university medical center, Nijmegen, The Netherlands; 7 Radboud Center for Infectious Diseases, Radboud university medical center, Nijmegen, The Netherlands

**Keywords:** Antibiotic stewardship, Quality indicator, Benchmarking, Antimicrobial stewardship team, Antimicrobial stewardship program, Quality of care

## Abstract

**Background:**

The Dutch Working Party on Antibiotic Policy is developing a national antimicrobial stewardship registry. This registry will report both the quality of antibiotic use in hospitals in the Netherlands and the stewardship activities employed. It is currently unclear which aspects of the quality of antibiotic use are monitored by antimicrobial stewardship teams (A-teams) and can be used as indicators for the stewardship registry. In this pilot study we aimed to determine which stewardship objectives are eligible for the envisioned registry.

**Methods:**

We performed an observational pilot study among five Dutch hospitals. We assessed which of the 14 validated stewardship objectives (11 process of care recommendations and 3 structure of care recommendations) the A-teams monitored and documented in individual patients. They provided, where possible, data to compute quality indicator (QI) performance scores in line with recently developed QIs to measure appropriate antibiotic use in hospitalized adults for the period of January 2015 through December 2015

**Results:**

All hospitals had a local antibiotic guideline describing recommended antimicrobial use. All A-teams monitored the performance of bedside consultations in *Staphylococcus aureus* bacteremia and the prescription of restricted antimicrobials. Documentation and reporting were the best for the use of restricted antimicrobials: 80% of the A-teams could report data. Lack of time and the absence of an electronic medical record system enabling documentation during the daily work flow were the main barriers hindering documentation and reporting.

**Conclusions:**

Five out of 11 stewardship objectives were actively monitored by A-teams. Without extra effort, 4 A-teams could report on the quality of use of restricted antibiotics. Therefore, this aspect of antibiotic use should be the starting point of the national antimicrobial stewardship registry. Our registry is expected to become a powerful tool to evaluate progress and impact of antimicrobial stewardship programs in hospitals.

## Background

Inappropriate use of antibiotics contributes to the growing problem of antimicrobial resistance, which is a serious threat to public health [1]. Hospital-based programs dedicated to improve antibiotic use, commonly referred to as “Antibiotic Stewardship Programs” (ASPs), aim to optimize the treatment of infections, reduce adverse events including antibiotic resistance, and to improve patient outcome [2]. To curb antimicrobial resistance, the Dutch Health Care Inspectorate and the Minister of Health have recommended the establishment of antimicrobial stewardship teams (A-teams) in every hospital. Since 2014, most Dutch hospitals have started the implementation of ASPs by A-teams.

To measure the progress and impact of the national implementation of antimicrobial stewardship, the Dutch Working Party on Antibiotic Policy (SWAB) aims to develop an antimicrobial stewardship registry. A registry is an organized system that uses observational study methods to collect uniform data to evaluate specified outcomes for a defined population and that serves one or more predetermined scientific, clinical, or policy purposes [3]. Quality focused registries -assessing quality of care and quality improvement- are increasingly created to measure differences in quality of care between providers or patient populations. These registries collect data in the form of process of care indicators [3].

Process indicators assess healthcare service delivered to patients by healthcare providers [4]. These data may be used to, for example, demonstrate opportunities for improvement, or provide transparency through public reporting.

The national antimicrobial stewardship registry will report on an annual basis both the quality of antibiotic use in hospitals in the Netherlands and the stewardship activities employed by the A-teams. In this manner, the establishment of a comprehensive and detailed prospective quality-focused registry continuously provides data on the progress and impact of implementation of ASPs in Dutch hospitals. These data will be published in Nethmap, the annual national report which currently reports on consumption of antimicrobial agents and on antimicrobial resistance patterns [5].

The value of clinical registries have been shown to improve quality of care in studies in patients with severe infections admitted to the hospital [6, 7], but also in patients treated in an ambulatory setting with antibiotics [8]. To our knowledge, our registry will be the first national, quality focused, registry for hospital antimicrobial use.

It is currently unclear which quality of antibiotic use aspects are monitored in daily practice by A-teams and can be used as indicators for the stewardship registry. The selection of these indicators requires balancing the goals of the registry with the desire to meet the needs of the providers. For example: data should be readily available for measurement and retrievable without undue burden, and providing useable and relevant information [3]. Ideally, these indicators are already integrated in the daily workflow of the providers. Therefore, we performed a pilot study to assess the availability of data on quality of antibiotic use currently monitored and documented by Dutch A-teams, which could be a starting point for the national registry.

## Methods

### Setting & study population

An observational pilot study was performed in 2 university and 3 non-university hospitals (one small hospital and two large teaching hospitals). These 5 hospitals were selected since they had operational, experienced A-teams. They were thought to be able to demonstrate the variety of ‘what can be monitored, documented and reported’ during daily practice in a non-study setting.

### Hospital and A-team characteristics

The composition and year of establishment of the A-teams, number of hospital beds, and the presence of an Electronic Medical Record System and/or Electronic Prescribing System were recorded.

### Monitoring, documenting and reporting recommended antimicrobial use: Stewardship objectives and QIs

We visited each hospital for a systematic assessment. The A-teams were scored on 14 predefined stewardship objectives (Table 1). Eleven of these objectives reflect processes of care at the individual patient level. We asked A-team members whether they monitored patients in line with each objective and whether they documented the appropriateness of antibiotic use in those patients (Table 2).

**Table 1:** Stewardship objectives reflecting processes and organization of care, and the corresponding quality indicators

Number	Stewardship objective, process of care recommendation	Corresponding quality indicator	
		Numerator description	Denominator description
1	Take 2 sets of blood cultures before starting antibiotic therapy	Number of patients in whom at least 2 sets of blood cultures were taken before systemic antibiotic therapy was started	Total number of patients who started with empirical systemic antibiotic therapy
2	Take cultures from suspected sites of infection	Number of patients in whom cultures from suspected sites of infections were taken within 24 h after the systemic antibiotics were started	Total number of patients who started with systemic antibiotic therapy
3	Prescribe empirical antibiotic therapy according to local guideline^a^	Number of patients who started with empirical systemic antibiotic therapy according to the national guideline	Total number of patients who started with empirical systemic antibiotic therapy
4	Adapt antibiotic dosage to renal function	Number of patients with a compromised renal function with a dosing regimen adjusted to renal function	Total number of patients who started with systemic antibiotic therapy which should be dosed according to renal function, and who had an unknown or compromised renal function.
5	Document antibiotic plan	Number of patients for whom an antibiotic plan was documented in the case notes	Total number of patients who started with systemic antibiotic therapy
6	Change empirical to pathogen-directed therapy	Number of patients with empirical therapy whose culture became positive and changing to pathogen-directed therapy was done correctly.	Total number of patients with empirical systemic antibiotics, whose culture became positive
7	Switch from intravenous to oral therapy on the basis of the clinical condition and when oral treatment is adequate	Number of patients with intravenous antibiotics for 48-72 h, in whom changing to oral antibiotic therapy on the basis of clinical conditions was done.	Total number of patients with intravenous antibiotics for 48-72 h, in whom changing to oral antibiotic therapy on the basis of the clinical condition was indicated
8	Perform therapeutic drug monitoring when the therapy is >3 days for aminoglycosides and >5 days for vancomycin	Number of patients on aminoglycosides or vancomycine in whom a serum drug level has been determined after respectively >3 or >5 days of therapy	Total number of patients who received aminoglycosides for >3 days and/or vancomycin for >5 days
9	Discontinue antibiotic therapy if infection is not confirmed	Number of patients whose empirical antibiotic therapy was discontinued within 7 days based on the lack of clinical and/or microbiological evidence of infection.	Total number of patients who started empirical systemic antibiotic therapy, but lacked clinical and/or microbiological evidence of infection.
10	Perform ID specialist bedside consultations in hospitalized patients with a *Staphylococcus aureus* bacteremia	Number of patients with *Staphylococcus aureus* bacteremia who had a bedside consultation of an ID specialist	Total number of patient with a *Staphylococcus aureus* bacteremia
11	Assess patients’ adherence	Number of patients adherent to the prescription’s instructions	Total number of patients with a prescription of antibiotics
	Stewardship objective, organization of care recommendation		
12	A local antibiotic guideline should be present and an update should be done every 3 years		
13	The local guidelines should correspond to the national antibiotic guidelines but deviate based on local resistance patterns		
14	A list of restricted antibiotics should be present		

^a^antibiotics on a list of “restricted” and “limited prescription” antimicrobial drugs

The numerator and denominator described in the third and fourth column were used to calculate quality indicator performance. [9]

**Table 2:** Example of monitoring, documentation and reporting of a stewardship objective

*Monitoring:* a local member of the A-team assesses empirically prescribed restricted antibiotics for accordance with the local guideline, using daily generated lists (*Stewardship objective 3,* Table 1 *).*
*Documentation:* The A-team member documents both the appropriate and inappropriate prescriptions, including the prescribing department.
*Reporting:* Each department receives an annual report about the quality of empirical use of restricted antibiotics. Quality indicator performance is presented as the percentage of appropriate prescriptions.

To assess their ability to report hospital specific data on the quality of antibiotic use, we explored what data were readily available to compute process indicator performance scores. Such scores can be described as a percentage between 0 and 100 where the numerator represents the number of patients in whom antibiotics are used as defined, and the denominator represents the eligible target population. Next, we collected these data to compute QI performance scores in line with the recently developed quality indicators to measure appropriate antibiotic use in hospitalized adults for the period of January 2015 through December 2015 [9].

Similarly, information on the 3 stewardship objectives reflecting organization of care was collected by asking whether the recommendations on the organization of care -as defined using the structure QIs- were met or not in each hospital. Additional local initiatives aimed at measuring the quality of antibiotic use were also taken into account.

All available data were collected and extracted in a uniform way. If an A-team was not able to provide data, barriers to registration were discussed during the site visit with a member of the A-team. QI performance scores were computed for all indicators that could be reported by two or more hospitals (Table 1). The comparison of the results between the hospitals was not the primary aim of this study. Instead, we focused on assessing the possibility to provide data about the quality of antibiotic use. For this study, no approval of a medical ethics committee was required, since it was part of quality control of drug utilization, observational in nature, data used for this study were already available in the electronic patient records, and data were provided and processed anonymously.

## Results

### Hospital and A-team characteristics

We included 5 hospitals: 2 academic teaching hospitals with approximately 1000 beds, 2 smaller teaching hospitals with approximately 500 beds, and 1 hospital with approximately 250 beds. The characteristics of the 5 A-teams are shown in Table 3. The 5 A-teams included in this study operated on a daily basis, except for the weekends.

**Table 3:** Characteristics of the participating hospitals and their A-teams

Hospital		A	B	C	D	E
Number of hospital beds		1002	953	268	554	543
A-team composition	Hospital pharmacist	+	+	+	+	+
	ID specialist	+	+	+	+	+
	Microbiologist	+	+	+	+	+
	Information technician	−	−	−	−	+
	Nurse	−	−	+	−	−
	Quality of care specialist	−	+	−	−	−
Year of establishment of A-team		2014	2015	2013	2014	2014
Total numbers of prescriptions documented		343	1824	1729	575	436
Electronic Medical Record Present		−	+	−	−	+^a^
Electronic Prescribing System Present		+	+	+	+	+

*Abbreviations*: *ID* infectious disease

^a^only partially: written patient record but laboratory and microbiology results are accessible via Electronic Medical Record

### Monitoring and documentation

Regarding the organization of care objectives, all 5 hospitals had a local antibiotic guideline that corresponded to the national guideline and had been updated at least every 3 years. All 5 hospitals had a list of antibiotics that were restricted or had limited indication according to the list published in The Antimicrobial Stewardship Practice Guide for the Netherlands [10]. Restricted antibiotics (such as carbapenems and glycopeptides) have been defined as drugs that only should be prescribed for microorganisms that are resistant to the usual drugs. Limited indication antibiotics are drugs that are indicated for some indications but should not be used in other situations (such as quinolones and third generation cephaloporins).

Only 2 hospitals used an electronic medical record system for documenting process of care objectives, which enabled automatic data extraction. The other hospitals had written patient files in combination with an electronic prescribing system. In those hospitals, a member of the A-team manually recorded data in a spreadsheet.

Of the 11 process of care objectives, 5 were monitored by at least one of the A-teams during the study period (Table 4). Only 2 objectives were monitored by all A-teams: [1] prescribe restricted antibiotics and limited prescription antibiotics according to local guideline, and [2] perform bedside consultations for *S. aureus* bacteremia (Table 4).

**Table 4:** Monitoring, documenting and reporting the quality of antibiotic use by A-teams

		Hospital					
Process of care recommendation	Activity	A	B	C	D	E	Total
Blood cultures taken?	Monitored	−	−	−	+	−	1/5 (20%)
	Documented	−	−	−	+	−	1/5 (20%)
	Reported	−	−	−	+	−	1/5 (20%)
Antibiotics prescribed according to local guideline?	Monitored	+	+	+	+	+	5/5 (100%)
	Documented	+	+	+	+	+	4/5 (80%)
	Reported	+	+	+	+	-^*^	1/5 (20%)
Therapy switched from intravenous to oral therapy?	Monitored	−	+	+	−	+	3/5 (60%)
	Documented	−	+	+	−	−	2/5 (40%)
	Reported	−	+	-^*^	−	−	1/5 (20%)
Therapeutic drug monitoring performed?	Monitored	−	+	+	+	+	4/5 (80%)
	Documented	−	+	−	−	−	1/5 (20%)
	Reported	−	+	−	−	−	1/5 (20%)
Bedside consultation performed for *S.aureus* bacteremia?	Monitored	+	+	+	+	+	5/5 (100%)
	Documented	−	+	−	−	−	1/5 (20%)
	Reported	−	+	−	−	−	1/5 (20%)
*Organization of care recommendation*							
Local antibiotic guideline is present		+	+	+	+	+	5/5 (100%)
Local guideline corresponds to national guideline		+	+	+	+	+	5/5 (100%)
List of restricted antibiotics is present		+	+	+	+	+	5/5 (100%)

In all hospitals, a daily list of patients on restricted antibiotics or limited prescription antibiotics was generated, either automatically in the Electronic Medical Record, or manually by the pharmacy department. The prescriptions were evaluated for appropriateness (i.e. in accordance with the local guideline) by a local A-team member and an advice to the prescriber was given when necessary. One of these 4 hospitals monitored restricted antibiotics during a four-month period, the others performed continuous monitoring in 2015 (prospective audit and feedback). One A-team reviewed all prescribed antibiotics for appropriateness instead of only restricted and limited prescription antibiotics.

Four out of 5 hospitals documented the appropriateness of the use of restricted antibiotics and limited prescription antibiotics. The appropriateness (appropriate, inappropriate, indeterminate) of the prescriptions was recorded either in the patient chart or in a spreadsheet.

Four hospitals monitored therapeutic drug monitoring (TDM) in patients treated with aminoglycosides or vancomycin. Only one hospital documented data about the numbers of patients with an indication for TDM and whether drug levels were determined in this population.

An iv-oral switch monitoring program was performed in 3 of 5 hospitals: after 48–72 h on intravenous (iv) antibiotic therapy a local member of the A-team determined whether a patient was eligible for iv to oral switch, based on predefined switch criteria [11]. Appropriateness was documented by 2 A-teams only (Table 4). During a three-month period in 2015, hospital B documented which patients could be safely switched to an oral formulation 48-72 h after initiation, but did not provide advice to the prescribing physician. A second chart review was done 2 days after the initial assessment in hospital B to determine which patients were switched without an intervention of the A-team. The purpose of that local pilot study was to determine the need for monitoring incorrect iv-oral switch at specific wards. This showed that a switch was possible in 35% of all patients treated intravenously, but that this was only performed in 47% of the eligible cases. Metronidazole and amoxicillin were the antibiotics most often incorrectly continued intravenously.

Hospital C documented the cases for which a switch was recommended and performed, but the denominator (the total number of patients eligible for switch) was not documented, making it impossible to determine the quality indicator performance in that hospital.

One A-team monitored and documented on a daily basis all culture results and other microbiological procedures performed in patients empirically treated with antibiotics.

All hospitals monitored bedside consultation by an ID specialist in patients with *S. aureus* bacteremia. Data regarding the adherence to this objective was available in one hospital.

The 2 most frequently mentioned barriers for the lack of documentation were lack of time and the absence of an electronic medical record system enabling the documentation during the daily work flow.

### Reporting

A total number of 4907 (range 343–1824) prescriptions were monitored and documented by the local A-teams in 2015. To compute QI performance scores in line with quality indicators to measure appropriate antibiotic use in hospitalized adults, both the numerator and the denominator had to be known. One A-team documented the numbers of guideline-adherent prescriptions only, but could not report the denominator (the total number of antibiotic prescriptions). Unfamiliarity with the purpose of process indicators (problem identification, evaluation of interventions, and benchmarking) was the main reason for this missing information.

**Fig. 1:**
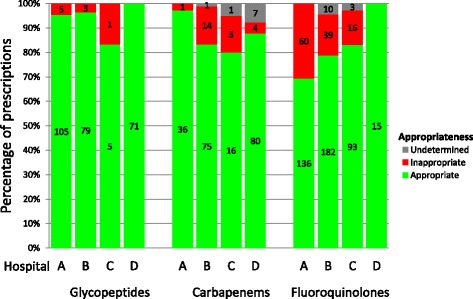
Appropriateness of antibiotic prescriptions. Number in the bars represents the numbers of prescriptions reviewed per category. In hospital “D” pre-authorisation for the use of glycopeptides resulted in an appropriateness of 100%

Comparing QI performance scores was possible for the appropriateness of glycopeptide, fluoroquinolone and carbapenem prescriptions for 4 hospitals (Fig. 1). Pre-authorisation by the medical microbiologist for glycopeptide prescriptions was mandatory in one hospital, which resulted in 100% appropriateness as judged by the A-team. Regarding the fluoroquinolones, 1 hospital (hospital D, Figure 1) only monitored the use of levofloxacin.

## Discussion

This observational pilot study investigated which stewardship objectives were monitored, documented and could be reported for the purpose of establishing a national antimicrobial stewardship registry. Our results showed that all A-teams have an antibiotic guideline available, and that monitoring is performed for only part of the recommended stewardship objectives. Furthermore, most A-teams have already implemented data collection and documentation for some of the appropriate antibiotic use aspects, enabling the measurement of QIs.

The initiative of the SWAB to establish an antimicrobial stewardship registry enables the measurement of the progress and impact of the national implementation of antimicrobial stewardship, and provides feedback to hospitals, which can be used to improve quality of antibiotic use in daily practice. This monitor should, ultimately, at least include the 14 stewardship objectives. From a viewpoint of data availability and feasibility, our results suggest that the antimicrobial stewardship registry should, firstly, focus on the appropriateness of the use of restricted antibiotics and limited prescription antibiotics. Additional QIs can be subsequently introduced into the monitor. It is important that new QIs should always be in line with the possibilities of the A-teams to provide data. Other aspects that should be taken into account when extending the monitor are the clinimetric properties of the QIs: measurability, applicability, inter-observer reliability, room for improvement and case mix stability. A recent observational multicenter study including 1890 patients receiving antibiotics for a suspected bacterial infection in 22 hospitals in the Netherlands concluded that seven of the QIs (which were also used in the present study) had sound clinimetric properties, whereas low applicability (i.e. QI applies to ≤10% of the reviewed patients) was found for the QIs ‘therapeutic drug monitoring’, ‘adapting antibiotics to renal function’ and ‘discontinue empirical therapy in case of lack of evidence of infection’ [12].

The optimal frequency of measuring quality of care is not known. Measurements can be performed daily, such as continuous prospective audit and feedback as performed in the present study, or during a certain time period (an audit), or by (repeated) point prevalence studies. Continuous measurements have the advantage that the quality of care for less common infections and infrequently used antibiotics can be measured, that trends can be easily visualized, and that results are less influenced by season, outbreaks or incidental errors. The biggest drawback, however, is its time-consuming nature. Buyle et al. collected data over a three-month period to assess the feasibility of a QI for intravenous to oral switch [13]. The indicator was measurable in 99.1% of cases, but the median time needed for case assessment and documentation was 29 min, which is not feasible in daily practice. A practical approach could be to replace continuous measurements with intermittent measurements if the QI performance score is sufficient.

Although systematic documentation of quality of antibiotic use was performed to some extent, it was not the main priority for A-teams. This is in line with previous hospital studies [14, 15]. We believe that standardized documentation of quality of antibiotic use is a prerequisite for the assessment of the quality of antibiotic use, providing valuable information: about the aspects of care that are most in need of improvement, the progress of implementation of an ASP and the effect of stewardship activities. The present study showed that only 10% of glycopeptide prescriptions were inappropriate, while inappropriate use of fluoroquinolones was common. This implies that identifying determinants for the misuse of fluoroquinolones and improving the quality of its prescription may be prioritized over monitoring other areas, such as glycopeptide use. Furthermore, the hospital performance may be used as a benchmark to gain insight into the national progress of implementation of ASPs and enables the exchange of best practices between hospitals.

Lack of time and the absence of a systematic and robust registration system were important barriers for documenting and therefore reporting the quality of antibiotic use. In three out of five hospitals, data were entered manually into a spreadsheet, which is prone to errors and leads to duplication of work. Nevertheless, although registration systems differed, we showed that 4 out of 5 hospitals could provide reliable data for benchmark and quality assessment.

The importance of integrating documentation into the daily work flow was supported by a recent study identifying barriers for the implementation of an antibiotic checklist [16]. Therefore, we strongly suggest that every A-team involves an information technician for data collection and management, and that hospital boards should support the introduction of an electronic medical record suitable for this purpose. To achieve these goals and to implement a functional ASP, appropriate financial support is needed, as shown by a recent French study [17]. A study of ASP costs is currently being performed in the Netherlands to help the A-teams allocate the resources for the implementation of ASPs, including the monitoring, documenting and reporting of the quality of antibiotic use.

Although only 5 hospitals were included in this pilot study, these varied from small non-university hospitals to large tertiary centres and can be regarded as representative of the Dutch hospital care setting. Besides investments in both human resources and ICT, all A-teams should be taught the value of documenting and reporting the quality of antibiotic use. Another important aspect that should be addressed is the use of uniform formats of data collection and the completeness of the local guidelines.

## Conclusion

Since 2014, the Dutch government requires all hospitals to establish an A-team. This observational study reports which aspects of quality of antibiotic use are monitored and documented by A-teams. At present, the quality of use of restricted and limited prescription antibiotics is the best documented ASP activity. In addition, this study has identified the need to standardize data collection as the basis for measuring and improving the quality of antibiotic use in hospitals and as input for the envisioned national antimicrobial stewardship registry.

## Abbreviations

ASPs: Antibiotic Stewardship Programs
A-teams: Antimicrobial stewardship teams
Iv: Intravenous
QI: Quality indicator
SWAB: Dutch Working Party on Antibiotic Policy
TDM: Therapeutic drug monitoring

## References

[1] Huttner A, Harbarth S, Carlet J, Cosgrove S, Goossens H, Holmes A (2013). Antimicrobial resistance: a global view from the 2013 world healthcare-associated infections forum. Antimicrob Resist Infect Control..

[2] Barlam TF, Cosgrove SE, Abbo LM, MacDougall C, Schuetz AN, Septimus EJ (2016). Implementing an antibiotic stewardship program: guidelines by the Infectious Diseases Society of America and the Society for Healthcare Epidemiology of America. Clin Infect Dis.

[3] Gliklich R, Dreyer N, Leavy M, Eds. Registries for evaluating patient outcomes: a User’s guide. Third edition. Two volumes. AHRQ publication no. 13(14)-EHC111. Rockville, MD: Agency for Healthcare Research and Quality. April 2014. http://www.effectivehealthcare.ahrq.gov/registries-guide-3.cfm. Accessed 19 May 2017.

[4] Donabedian A (1988). The quality of care. How can it be assessed?. JAMA.

[5] SWAB. NethMap (2016). Consumption of antimicrobial agents and antimicrobial resistance among medically important bacteria in the Netherlands in 2015.

[6] Kumar A, Roberts D, Wood KE, Light B, Parrillo JE, Sharma S (2006). Duration of hypotension before initiation of effective antimicrobial therapy is the critical determinant of survival in human septic shock. Crit Care Med.

[7] Martin CM, Priestap F, Fisher H, Fowler RA, Heyland DK, Keenan SP (2009). A prospective, observational registry of patients with severe sepsis: the Canadian sepsis treatment and response registry. Crit Care Med.

[8] Nathwani D, Tice A (2002). Ambulatory antimicrobial use: the value of an outcomes registry. J Antimicrob Chemother.

[9] van den Bosch CM, Geerlings SE, Natsch S, Prins JM, Hulscher ME (2015). Quality indicators to measure appropriate antibiotic use in hospitalized adults. Clin Infect Dis.

[10] SWAB. The antimicrobial stewardship practice guide for the Netherlands; 2015. Available online at: http://www.ateams.nl/de-praktijkgids/download-de-praktijkgids. Accessed 16 Nov 2016.

[11] Sevinc F, Prins JM, Koopmans RP, Langendijk PN, Bossuyt PM, Dankert J (1999). Early switch from intravenous to oral antibiotics: guidelines and implementation in a large teaching hospital. J Antimicrob Chemother.

[12] van den Bosch CM, Hulscher ME, Natsch S, Wille J, Prins JM, Geerlings SE. Applicability of generic quality indicators for appropriate antibiotic use in daily hospital practice: a cross-sectional point-prevalence multicenter study. Clin Microbiol Infect. 2016;22(10):888.e1–888.e9 10.1016/j.cmi.2016.07.011 27432770

[13] Buyle FM, Metz-Gercek S, Mechtler R, Kern WV, Robays H, Vogelaers D (2012). Prospective multicentre feasibility study of a quality of care indicator for intravenous to oral switch therapy with highly bioavailable antibiotics. J Antimicrob Chemother.

[14] van Limburg M, Sinha B, Lo-Ten-Foe JR, van Gemert-Pijnen JE (2014). Evaluation of early implementations of antibiotic stewardship program initiatives in nine Dutch hospitals. Antimicrob Resist Infect Control.

[15] Pollack LA, van Santen KL, Weiner LM, Dudeck MA, Edwards JR, Srinivasan A (2016). Antibiotic stewardship programs in U.S. acute care hospitals: findings from the 2014 National Healthcare Safety Network Annual Hospital Survey. Clin Infect Dis.

[16] van Daalen FV, Geerlings SE, Prins JM, Hulscher ME (2016). A survey to identify barriers of implementing an antibiotic checklist. Eur J Clin Microbiol Infect Dis.

[17] Le Coz P, Carlet J, Roblot F, Pulcini C (2016). Human resources needed to perform antimicrobial stewardship teams’ activities in French hospitals. Med Mal Infect.

